# Event and Comment

**Published:** 1909-04-15

**Authors:** 


### DENTAL REGISTER.

**Vol. LXIII. April 15, 1909. No. 4.**

### EVENT AND COMMENT.

FLETCHERISM. We devote considerable space in this issue to the presentation of two phases of a subject which is engaging the attention of many people at the present time, and which should be of the greatest concern to the Dental Profession,—the so-called "Fletcherization" of food. We have wanted to present this matter to our readers for some time but have not had the proper material until now, and we trust all who may read these two addresses shall be as fully impressed with their value to humanity and their peculiar interest to the Dental Profession that we believe them to be. The one appeals strongly to our altruistic interests, and the other equally strongly to the development of a higher sense of the importance of our calling. It seems strange that a layman should have been called to show us the very great importance and even sacredness of our calling. It is true that every earnest practitioner of long experience comes to realize the value of the teeth and strives more and more to keep them in good functional condition, but it seems that few writers and practitioners have attained the heights of appreciating the fundamental fact that every tooth is in a sense invaluable and should be kept in perfect form and health as well as in its proper position in the dental arches so that adequate function may be made possible. The great emphasis Mr. Fletcher puts upon the proper mastication of food in the mouth rather than in the stomach and intestines, because of its sanitary effect or influence so far as body health and ability to work is concerned, in itself ought to be sufficient to cause us to renew our efforts to place the dental organs in the best possible condition when crippled by disease, and should stimulate anew in us the effort to prevent the destroying influences that are constantly encroaching upon these vital organs. The economic waste is no doubt sufficiently important to command our best services, but as Mr. Fletcher has so ably indicated our duty in the moral realm is equally insistent. We have often heard it said that intemperance and immorality were largely due to bad cooking and slovenly housekeeping; but it has never occurred to us that bad dentistry was in any way associated with depravity in morals and good citizenship. We have sometimes heard that insanity could be produced from disease of the teeth, but no one believes that it is a common or even frequent cause of this dreadful disease; likewise appendicitis and many other specific diseases have been traced for incipient cause to the mouth and teeth. And now we are confronted with the alarming hypothesis that bad mastication of food, which of course in most cases is due to bad teeth, is responsible for most of our physical and moral ill health. It seems that many things are just now conspiring to bring our profession to a realization of this startling propaganda, and we trust that the great work of our modern orthodontists and oral prophylaxis adherents shall have prepared the way and means for practically testing out this new hypothesis and of proving its accuracy. If Mr. Fletcher shall succeed in convincing the people of the value of a tooth, and the profession that every tooth in every individual is worth his best efforts in preservation, he will certainly have accomplished a work worthy of the efforts of any man.

**FLETCHER'S KINDERGARTEN OF VITAL ECONOMICS.** The East Side, that curio shop of odd institutions, is having an addition made to its collections in the form of a Kindergarten of Vital Economics, which in a few weeks will be opened at 325 East Thirty-first street.

On the fifth floor of a model tenement Horace Fletcher, the man who proposes to revolutionize the gentle art of eating in the interest of health and happiness, is superintending workmen who are transforming a five-room apartment into quarters for what is to be a cross between a clinic and a training school.

Partitions have been torn out, making a lecture room large enough for a hundred seats. Three large windows opening to the south, together with the delicately tinted walls give the place a cheerful aspect, while the presence of tubs, a gas stove and a sink, in what was once a kitchen, add a touch of realism which recalls one to the exigencies of life on the east side.

A small room opening off the lecture hall is to serve as the director's office. Here Mr. Fletcher will keep the records of his pupils and will bestow the personal advice which many cases will need. Here, too, will be installed the apparatus for testing endurance by which the progress of the students will be gauged.

Assisting Mr. Fletcher in this end of the work is John F. Stapleton, physical director of St. Luke's School in Philadelphia, who for five years has been an enthusiastic Fletcherite and whose fame as a strong man has added no small prestige to the Fletcher movement.

In his apartment on the floor above Mr. Fletcher, whose permanent home is on the Grand Canal in Venice, but who prefers a Phipps tenement to the Waldorf-Astoria as a New York dwelling place, discourses cheerfully on his plans and his discoveries. He is a white-haired gentleman who has safely survived his three-score years. He is small of stature and ruddy of cheek. He moves with a quietness which eliminates all suggestion of nervous haste, and speaks in a voice which evidences kindliness and culture.

The mission-furnished study where Mr. Fletcher receives his guests is the retreat of a worker. The large pile of mail on his desk indicates the wide interest he is awakening. Unlimited books and papers, proofs of lecture announcements and half-finished aticles are evidence of how the apostle of simplified eating spends his time.

Mr. Fletcher is in every way a delightful man to interview, from the cordiality with which he invites one to a Fletcher luncheon to the enthusiasm with which he talks about his work; but for all that he is a disappointment.

A man who has the audacity to accuse the civilized world of not knowing how to eat and who is attempting to revolutionize housekeeping and to bisect the food supply ought to be a fanatic. That, of course, is the least one expects. But after futile attempts to extract incriminating evidence one is forced to pronounce the verdict of not guilty.

Mr. Fletcher hasn't an ounce of dogmatism about him. He believes, above all else, in keeping the open mind. What is more, he refuses to take the serious attitude of the typical reformer. He not only approves of having a good time in any way that offers, but he is for turning work into play.

His views on eating are very simple and altogether lacking in his aggressiveness which ought to be his stock in trade. He isn't even a vegetarian. He doesn't proscribe the pies of one's grandmother. He offers no elaborate theories on the chemistry of food. He refuses to make out a table of weights and measures. Eat whatever you please, says Mr. Fletcher, only eat it right, and don't overcrowd your system.

What is more, while he firmly believes that a perfect set of beings will be the corollary of Fletcherism, he doesn't find everything all wrong under the present system. Even when he talks most enthusiastically about his work he offers the proof before the theory.

He tells you that ten years ago when he was low in health, he discovered that by eating very slowly and chewing minutely he didn't need nearly as much food, he improved in health and he made enormous gains in physical strength. He goes on to explain how he investigated this discovery and found that man had drifted into his present habit of eating by accident.

It is a far cry from the meagre fare of the cave to the twenty course dinner. Once man ate because his appetite commanded him. Now it is a habit to seek a well-laden board three times a day, which is almost as binding as the Ten Commandments.

What is more, eating has become a useful device for killing time and spending money. Food is abundant and cheap, and people eat in quantities, not because they are hungry but because it is the thing to do. As a consequence the organs are overworked, and the unassimilated food forms poisons.

All of which has a plausible sound, revolutionary though it is. When Fletcher announced his discovery no one believed him. When he offered proof men of science took him by the hand, but the world at large turned him over to the funny papers. Ten years have passed now, and gradually a body of 200,000 converts who have tested the theory and found it good have changed derision to serious interest.

It is at this point that Mr. Fletcher, who has formerly spread his propaganda merely by his pen, has come to New York to inaugurate a definite campaign. His beginning is small and tentative. He thinks it much wiser to emulate the spirit of growth than it is to start the school on a grand scale. The school Mr. Fletcher intends to keep small so that it will have a big future.

The immediate ends for which the Kindergarten of Vital Economics is working are to spread the propaganda among teachers and social workers who come in contact with masses of people and to work directly with the people of the East Side, who are most in need of the new gospel of nutrition. The school is not to be a charity. On the other hand, no set tuition will be charged.

Mr. Fletcher is confident that people who can afford to pay will without being asked contribute enough for the running expenses of the school. That end of the enterprise is causing him no anxiety. Nor is he worried as to where his pupils are coming from.

Already a class of eighteen volunteers who are making notes on their present habits and conditions is waiting for the doors to open. Large numbers from Fifth avenue and the park districts have asked to be enrolled as pupils. Mr. Fletcher declares that the Kindergarten of Vital Economics is to be a democratic institution which makes its selection of the basis of need only and bars out neither rich nor poor.

But as the Fletcher system involves a saving of money as well as an increase of health to people with a limited purse it will fulfill a double mission.

What Mr. Fletcher proposes to do for his pupils is to teach them to get control of their bodies as a chauffeur is taught to manage a 90-horsepower automobile. Any physical, mental or moral disability means bad management.

The pupils will be put through an examination on entering the class. Among other things their physical endurance will be carefully tested. Not until they become models of their own possibilities will they be sent forth as graduates.

How long this will take will depend on the individual case; also the amount of instruction will vary with the individual. Some pupils will probably increase their physical endurance 100 per cent., dispose of their ailments and become optimistic altruists in a month or two. Others will take much longer.

Mr. Fletcher himself, when he dropped into the Yale gymnasium last summer, raised 300 pounds 350 times, while the record of the best athlete was 175 times. It was by taking the training of the Yale crew on an 11-cent-a-day fare that he first won recognition five years ago.

The luncheon to which the reporter was invited to stay was as surprising as the host. It was not at all the bread and water of the ascetic. Instead the party of three sat down to a daintily appointed table where fried bananas, creamed asparagus, toast, chocolate, sliced oranges, nuts and candies were served.

One o'clock though it was, Mr. Fletcher had not eaten since the night before and he had been up and at work since 6. It may have been the Fletcher spirit of the place, possibly the leisurely pace and the entertaining conversation but one found oneself refusing a second helping for fear of overeating at a Fletcher table.

Mr. Fletcher explained that it was a warmed-over meal left from a luncheon party of the day before. According to the Fletcher system there is no waste. What isn't eaten one time is saved for the next meal.

When the cost of the luncheon was counted up it proved to be 19 cents for three. The average price of meals since Mr. Fletcher set up his Menage has been 15 cents a meal, which with two meals a day demands a 30-cent food allowance. This, Mr. Fletcher explained, was coupled with no effort at economy, for he was living on the fat of the land.—*New York Sun.*

